# Perspectives on treatment side effects in patients with metastatic gastrointestinal stromal tumour: a qualitative study

**DOI:** 10.1186/s13569-019-0116-3

**Published:** 2019-04-30

**Authors:** Lena Fauske, Ivar Hompland, Geir Lorem, Hilde Bondevik, Øyvind S. Bruland

**Affiliations:** 1 Department of Oncology, Norwegian Radium Hospital, Oslo University Hospital, P.O. Box 5960, Nydalen, 0424 Oslo, Norway; 2 Department of Interdisciplinary Health Sciences, Institute of Health and Society, University of Oslo, Postboks 1089, Blindern, 0317 Oslo, Norway; 3 Department of Health and Care Sciences, Faculty of Health Sciences, UiT The Arctic University of Norway, Hansine Hansens veg 18, 9019 Tromsø, Norway; 4 Institute of Clinical Medicine, University of Oslo, Oslo, Norway

**Keywords:** Gastrointestinal stromal tumour (GIST), Metastatic cancer, Side effects, Qualitative research

## Abstract

**Background:**

This study aims to explore how patients with metastatic gastrointestinal stromal tumour (GIST) experience the adverse effects of treatment, as expressed by the individuals themselves.

**Methods:**

A qualitative, phenomenological and hermeneutic design was applied. Twenty patients with metastatic GIST participated in the study. In-depth and semi-structured interviews were conducted and then analysed by means of an inductive thematic analysis.

**Results:**

The majority of participants reported experiencing a changed life after being diagnosed with metastatic GIST and commencing systemic medical treatment. More than half of them described partially debilitating self-reported side effects and complaints that had a detrimental impact on their lives. The life-prolonging tyrosine kinase inhibitor treatment prompted the participants to adapt to ‘a new normal’. Several participants also emphasised having an ambivalent relationship with the pill, although most looked upon it as ‘a friend’ because it kept them alive. Paradoxically, while the participants struggled with the side effects of treatment as well as the consequences of living with a chronic cancer, half of them considered themselves to be healthy and, thus, to not actually be cancer patients.

**Conclusions:**

We observed a gap between the biomedical perspective on disease that health professionals typically adopt and the individual experiences of patients living with metastatic GIST. For those patients who are living in limbo between having metastatic cancer and offered an effective treatment, a holistic view of health on the part of their healthcare providers seems crucial. A vital goal should hence be to improve communication between healthcare professionals and GIST patients so as to secure an individualised follow-up with guidance on coping with, and adapting to, their new normal.

*Trial registration* The study was approved by the data protection officer of the Oslo University Hospital (Approval Number 2016/15358)

**Electronic supplementary material:**

The online version of this article (10.1186/s13569-019-0116-3) contains supplementary material, which is available to authorized users.

## Background

Recent progress in the field of medical oncology has increasingly rendered cancer a chronic disease, with metastatic gastrointestinal stromal tumours (GIST) being a prime example of this phenomenon. Due to the seminal discovery of *KIT* mutations in cases of GIST [1], alongside the subsequent introduction of the tyrosine kinase inhibitor (TKI) imatinib [2, 3], metastatic GIST has changed from being a highly aggressive type of cancer that caused the death of almost all patients within the first year of diagnosis [4] to being a chronic cancer with a median overall survival rate of approximately 7 years [5]. Indeed, imatinib and the other TKIs that have been introduced as effective treatments for metastatic GISTs induce long-term remission in the majority of patients and, for some, even result in an extended life expectancy of decades [5, 6]. However, although imatinib has revolutionised the treatment of metastatic GIST, most patients will eventually experience drug resistance [3]. This is particularly true in cases of treatment with second- [7] and third-line TKIs.

Imatinib is taken orally on a daily basis, and it is considered to be moderately to well tolerated, at least when compared to conventional chemotherapy [8]. Although severe adverse effects are uncommon, virtually all patients treated with imatinib report some side effects, with the most frequent being anaemia, periorbital oedema and watery eyes, diarrhoea, muscle cramps (typically in the hands and legs), fatigue and nausea [3, 9].

In addition to the well-known medical side effects of imatinib, several practical and psychosocial challenges may influence the daily lives of patients, although the extent to which this is the case has not yet been well studied. For instance, as most patients with metastatic GIST eventually will succumb to their disease [6, 10], the fear of disease progression is undeniably a challenge for patients and their families [11].

Further, in one study, the prevalence of severe fatigue among 61 GIST patients who were receiving TKI treatment was found to be significantly higher (30%) when compared to the matched healthy controls (15%) [12]. The fatigued patients reported a lower quality of life (QoL) as well as increased impairment in all the functional domains, including psychological distress and physical functioning. Another study described the extended lifetime that results from the TKI treatment of GIST as being akin to a Sword of Damocles [11]. The patients reported a good global QoL, although the majority also reported a considerable fear of disease progression. They experienced significantly higher levels of psychological distress, functional impairments and difficulty making plans for the future [11].

Being ill as a result of a serious disease not only affects a part of an individual’s body or an organ, but also impacts his/her practical, social, intellectual and emotional needs. To the best of our knowledge, only one study concerning GIST that had a qualitative design (mixed methods) has previously been conducted [13]. That study emphasised that patients with metastatic GIST shared similar emotional journeys. The patients were found to experience five stages of disease management, namely crisis, hope, adaptation, ‘new normal’ and uncertainty. This entire process was found to have a detrimental impact on their lives [13].

In the current study, our aim was to explore how patients with metastatic GIST experience both living with their disease and the adverse effects of its treatment. By adopting a qualitative method involving a phenomenological approach that utilised an explanatory design, we aimed to better understand how patients voice their experiences.

## Methods

This study adopts a psychosocial and sociocultural perspective on health and illness in order to identify the reasons behind the experienced phenomena, as expressed by the participants themselves. In line with the study’s methodological framework and research questions, we have applied a phenomenological experience-based and hermeneutics interpretation-based approach to disease and illness [14, 15]. Phenomenological research aims to investigate individual human experiences (phenomena) as they manifest in both daily life and specific situations [16], while hermeneutics relates to the methods developed for achieving an understanding and interpretation of phenomena in a comprehensible manner [17]. Here, comprehension develops through the entire process, and it is based on the participant’s and the researcher’s pre-understandings, as well as on the historical and cultural contexts.

### Patients

Patients with metastatic GIST who were treated at the Department of Oncology, Norwegian Radium Hospital, Oslo University Hospital (NRH OUH), were included in this qualitative study. They were identified from the prospective sarcoma registry at the NRH OUH and their clinical data were extracted from that database. The inclusion criteria were: (i) confirmed metastatic GIST, (ii) receiving treatment with TKIs for at least 2 years prior to inclusion in the study and (iii) stable disease, which was defined as no change in the administration of the TKI. The study cohort consisted of 20 patients, 11 women and nine men, with a median age of 61 years (range 36–85). They all did write and speak Norwegian and were all undergoing regular follow-up at our sarcoma outpatient clinics. In the results section, the participants are identified by their gender and age. Demographic and clinical information concerning the participants is presented in Table 1. Twelve of the participants received the conventional 400 mg dose of imatinib, four had reduction of the dose to 200 mg due to adverse events, whereas two had dose escalation up to 800 mg due to disease progression on 400 mg. Two participants received sunitinib.

**Table 1:** Baseline demographic and clinical data concerning the study cohort

Total number of patients	20
Age (years)^a^	61 (36–85)
Sex	
Female	11 (55)
Male	9 (45)
Relationship status	
Married	9 (45)
Cohabiting	3 (15)
Single	8 (40)
Children	
Yes	16 (80)
No	4 (20)
Time since primary diagnosis (years)^a^	8 (2–22)
Time receiving systemic treatment (years)^a^	6 (2–15)
Primary tumour localisation	
Stomach	12 (60)
Small bowel	7 (35)
Rectum	1 (5)
*KIT/PDGFRA* mutations	
* KIT* exon 11	11 (55)
* KIT* exon 9	3 (15)
* KIT* exon 13	1 (5)
* PDGFRA* exon 18	1 (5)
* PDGFRA* exon 12	1 (5)
Not detected	3 (15)
Metastatic site	
Liver	10 (50)
Peritoneal	8 (40)
Liver and peritoneal	2 (10)
Metastasis at diagnosis	
Yes	10 (50)
No	10 (50)
Previous adjuvant imatinib treatment (12–36 months)	
Yes	5 (25)
No	15 (75)
Systemic treatment in a metastatic setting	
Imatinib	18 (90)
Sunitinib	2 (10)
Number of surgeries (including surgery to the primary tumour)	
No surgery	1 (5)
1	10 (50)
2	8 (40)
5	1 (5)
Radiotherapy	
Yes	2 (10)
No	18 (90)

The values given in parentheses are percentages unless otherwise indicated, ^a^values are the median (range)

*PDGFRA* platelet-derived growth factor α-gene

### Procedure

The recruitment of participants was performed by the treating oncologists at the NRH OUH. The first author provided detailed information regarding all the relevant aspects of study participation and also conducted the interviews. The average length of the interviews was 45 min (16 to 82 min). Sixteen interviews were conducted in relation to a previously scheduled routine clinical follow-up appointment at the NRH OUH, while four were conducted in the participants’ home. The interviews followed a semi-structured guide and they were conducted on a face-to-face basis. The interviews were transcribed verbatim by a medical secretary. The interview guide invited the participants to narrate their whole story from the time of diagnosis to the present day, and it included the following main topics: How do the disease and its treatment affect your daily life? How is your relationship with the pill? Do you consider yourself to be healthy or ill? (Additional file 1). All the collected information was stored confidentially, and thematic analyses were conducted on anonymised transcripts.

The study was approved by the data protection officer of the NRH OUH (Approval Number 2016/15358), and written informed consent was obtained from all participants prior to beginning the study.

### Data analysis

A thematic analysis is a qualitative approach that has been widely applied across the social, behavioural and applied health sciences [18]. The purpose of this method is to identify patterns of meaning across a dataset in order to answer the research question being addressed. The patterns are identified through a process of data familiarisation and data coding, as well as through theme development and revision. In this study, the entire dataset was coded in detail (i.e. a thorough, inclusive and extensive coding approach) by hand by the first author, as well as partly by the third and fourth authors. The codes were then divided into themes and concepts. The emergent themes formed the core of the analysis, and they were reflected upon in accordance with the study’s objectives and also compared with the existing literature and theory [18]. Throughout the entire process of analysis, the researcher regularly returned to the original data to check the themes and quotes, as well as to ensure that the meaning had not been lost during either interpretation or translation [19].

## Results

The majority of participants reported experiencing a changed life after being diagnosed with a GIST and beginning systemic treatment. The crucial life-prolonging TKI treatment had side effects that influenced their daily lives in negative and challenging ways, which caused the participants to adapt to ‘a new normal’. Several participants emphasised that they had an ambivalent relationship with the pill, although most looked upon it as ‘a friend’ because it kept them alive. Paradoxically, while the patients struggled with both the side effects of treatment and the consequences of living with a chronic cancer, half of them considered themselves to be healthy and, thus, to not actually be cancer patients. We identified two dominant themes, namely self-reported adverse treatment effects and the paradoxical self.

### Self-reported adverse treatment effects

The participants reported varying degrees of self-reported side effects stemming from imatinib and sunitinib, although their subjective experiences of those side effects and their everyday consequences were similar in many ways. In addition to the side effects of taking daily medication, several participants pointed out that the gastrointestinal surgery had also resulted in challenges, such as abdominal pain, dumping syndrome or food intolerance.

**Fig. 1:**
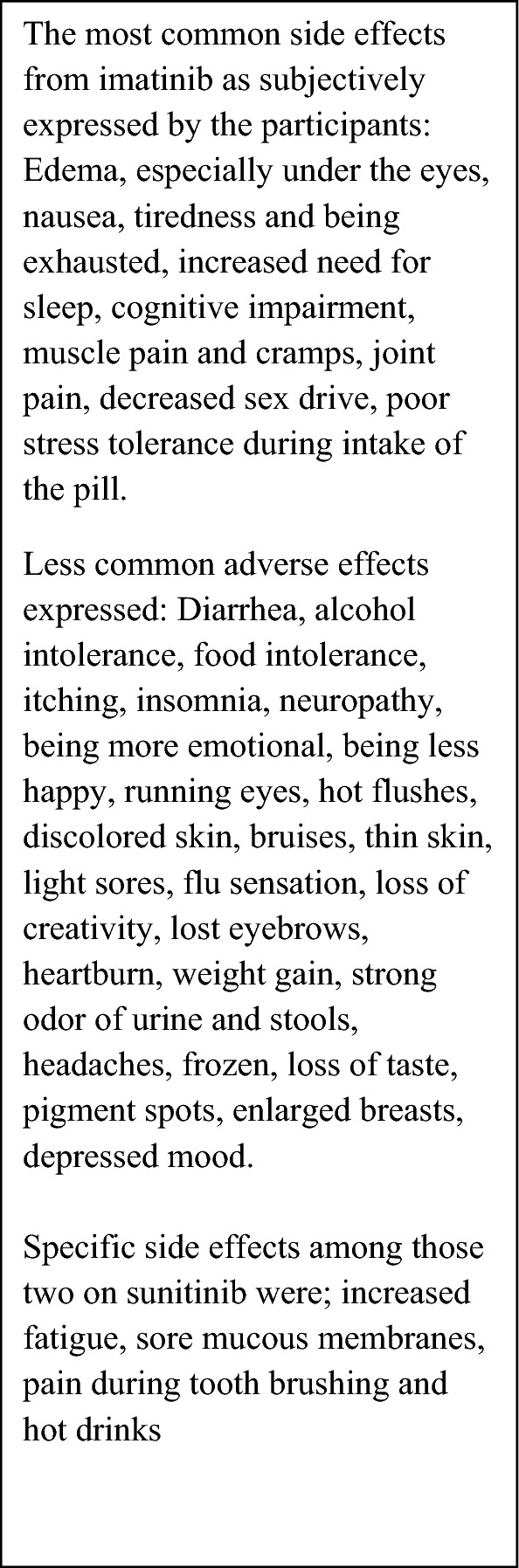
Self-reported subjective side effects

The majority of participants mentioned the well-known side effects of imatinib, such as oedema, especially periorbital oedema, nausea, diarrhoea, muscle cramps, muscle aches, joint pain, tiredness and exhaustion. Many also reported experiencing an increased need for sleep, cognitive challenges, reduced sexual desire, as well as poor stress tolerance around the intake of the pill. Furthermore, some mentioned alcohol and certain food intolerances, itching, insomnia and neuropathy, among other less common side effects (Fig. 1).

Based on their stories, the participants were divided into three groups. In the first group were four participants who had few self-reported side effects and complaints, which had only a minor influence on their everyday life, and who lived almost as they did before. In the second group were five participants who had some self-reported side effects and complaints, which meant that they experienced limitations and the need to adjust, although their everyday life was reasonably satisfactory. In the third group were 11 participants who reported extensive, partially debilitating, self-reported side effects and complaints that had a detrimental influence on their lives.

As mentioned above, more than half of the participants reported that the experienced side effects had a considerable negative impact on their daily lives. For instance, one woman (age 52) experienced the effect of a daily dose of 400 mg of imatinib as terrible:

‘I had cramps in all the muscles all over my body […] and with a lot of oedema in my face and legs [...] I lost two litres of fluid every morning. [...] I also got enlarged breasts […] they went up roughly one size a year. [...] And I needed a lot of sleep too, I felt fatigued.’

She also stated that the medicine reduced her wellbeing, although it did have a positive side: ‘It’s awful that there are so many side effects, but then I think it has helped me.’ She reduced her dose to 200 mg after a few years, which led to fewer side effects and a better life. Several others reported that reducing the medication dose led to a reduction in complaints. Further, the side effects were found to change over time, for example, nausea and cramps might decrease, while tiredness might increase. One male participant (47) said:

‘As time has passed, I’ve noticed that I’m a bit weaker in my whole body, more tired especially, so I go to bed a bit earlier than usual. It seems to be a feeling like the one I normally have when getting the flu [...], then you feel a bit shivery, in fact it’s a kind of normal state. But I think it has come on gradually, I don’t think it was like that in the beginning, during the first few years.’

Furthermore, several participants stated that their tiredness had become more pronounced after they had been taking imatinib for some years.

Many participants reported having become used to the side effects and having learned to adapt their lives accordingly. However, this was not the case for the two participants who were on a higher dose (800 mg daily). They reported many side effects, several of which were exhausting, debilitating and had a profound negative effect on their lives. For instance, one woman (49) stated that the medicine had such strong side effects that she lost all her energy, had a lot of cramps, felt cold and tired all the time, and did not dare to meet people, so she just stayed indoors. ‘My skin... swollen mucous membranes […] people could hardly recognise me, I developed a huge face, you could hardly see my eyes. […] It just developed and got worse and worse and worse.’ She stressed that she had been unprepared for such changes: “I didn’t think that this medicine would completely knock me out.’

Sunitinib is the first choice of alternative treatment when resistance to imatinib occurs [8]. The two participants who took sunitinib described more and stronger side effects from that drug when compared to what they experienced when they took imatinib. Both reported that tiredness and exhaustion were more of a challenge than before. One male participant (52) described pain in his skin and mucous membranes:

‘And physically, I can feel the side effect that I call sunburn under the skin. […] I get strong side effects if I drink alcohol. I’ve stopped liking coffee, tea and hot drinks because my tongue’s very sensitive. […] I don’t drink so many fizzy drinks now because getting those bubbles on my tongue is like getting barbed wire on my tongue. It’s a real drag, the sore feeling in my skin and hands, and inside all over my body, and in my mouth and mucous membranes, and it hurts just to clean my teeth. It really hurts. So, my dental hygiene isn’t so good now. Just try to brush your teeth with a steel brush. […] And the toothpaste tastes like strong whiskey. […] When I use sunitinib, all those side effects are very powerful.’

Many participants mentioned the rapid effect of treatment after starting imatinib, with the pain related to the disease being reduced after just a few days. They were grateful that there was a pill that could reverse the disease, or at least keep it under control, so that they could live with metastatic GIST. However, both the imatinib and sunitinib patients stated that there were two sides to the coin: ‘It’s both a friend and an enemy - without it I have no life. That’s simple maths,’ said a male participant (52). He also said that the pill was a daily reminder that he had a serious illness, which he otherwise tried not to focus on. It extended his life for several more years, but it also led to him experiencing many side effects and challenges. The majority of participants looked upon the pill as a friend because it kept them alive, although they emphasised a certain level of ambivalence in relation to it due to all the side effects. Despite the latter, none of the participants had ever considered stopping taking the drug.

### The paradoxical self

Despite having metastatic GISTs, many of the participants did not think of themselves as being cancer patients in their everyday lives. Nevertheless, they emphasised how their daily dose of medicine and its side effects regularly reminded them of their condition. However, they continued to feel healthy in their daily lives and, most of the time, they hence did not think about their cancer. For instance, a young man (36), whose side effects were so minimal that he could continue to work, stated:

‘Basically, I consider myself to be healthy, but obviously I’ve started to understand now, in the last six months, or the last year, that I’m still having treatment, so it’s a kind of in-between thing in a way. But in my day-to-day life, I’m healthy. [...] As long as I can do the things I like doing, I’m content.’

Being healthy was often not voiced as being related to a lack of illness, but rather to doing things that were important to the participants. One man (73) said, ‘I consider myself to be healthy. I really do. Because I want to do things, I like being outdoors, in the woods, picking berries and so on, that’s what I’ve done all my life.’ Being healthy was related to being able to live a normal life. The participants wanted to make the best of their lives and to not focus on cancer. One woman (74) said, ‘I’m basically healthy. So, I try to live as normally as I possibly can.’ Even some of those participants who did view themselves as cancer patients did not generally talk about their disease. One woman (51) who experienced many side effects of imatinib said:

‘That’s life. […] But the most important thing is to make an effort and to try to live as normally as possible in spite of … [cancer]. […] Having a job, that’s really important. Don’t just sit at home thinking and brooding, but do everything you used to do. […] Maybe you’ll need some extra breaks, well, just take them then.’

Several participants mentioned having made a conscious choice not to focus on their illness and all the associated negatives. One woman (52) stressed, “Yes, I changed my focus. Of course, sometimes I feel a bit down and then I just sit down at home and cry a few tears. And then I think “change your focus”. I prefer just to think I am not ill.’ An elderly woman (85) with a serious exhaustion problem said, “When I feel a depression coming on and I’m very tired and I’ve got no energy, then I say, “You have no right to feel like this, you’ve just got no right”. And then it passes.’ Some participants pointed out that categorising themselves as healthy despite their cancer did not necessarily mean that they were repressing the disease; instead, it related to the role they wanted to play. One woman (51) who experienced severe side effects stressed that she did not want to consider herself to be ill: ‘No, so I see myself as healthy. Because I don’t want to be ill.’ Several others pointed out that they managed to focus on the healthy and normal aspects of their life, which enabled them to live positively despite the cancer and the side effects of treatment.

## Discussion

This study examined patients’ experiences living with GIST and undergoing TKI treatment, as well as how the experienced adverse effects significantly challenged their daily lives. The predominant patient narrative concerned living with treatment and how it was established as part of ‘a new normal’. More than half of the participants in the current study described how extensive, partially debilitating, self-reported side effects and complaints had a detrimental influence on their lives. Interestingly, none of the participants questioned their medical treatment. The reason for this might be that the participants considered the life-prolonging effect of TKI treatment to outweigh the adverse effects. Our interpretation of a paradoxical self may relate to the fact that participants who were dependent on life-prolonging cancer treatment and who experienced adverse effects still considered themselves to be healthy. However, their experiences with imatinib or sunitinib, as treatments for metastatic GISTs, altered their self-identity and what good health meant to them. Many participants emphasised that being healthy and normal meant being able to still do what they could do before, for example, going to work or being busy with something that was enjoyable or important to them.

By conducting in-depth interviews with patients living with metastatic GIST, we found that more than half of them told a story that did not comply with the prevailing biomedical comprehension of the imatinib toxicity profile. Their subjective experience with side effects of treatment had significant physical, practical and social consequences for their everyday life. Hence, we observed a gap between the biomedical perspective on disease that health professionals typically adopt and the individual lived experiences of patients with metastatic GIST. Understandably, the biomedical approach of health professionals and the pharmaceutical industry alike is more concerned with the objective medical side effects of a treatment than with its overall impact on patients’ daily lives. The biomedical model could be described as reductive and, thus, criticised for being too narrow. It is a model that primarily focuses on cure or extended survival but it says next to nothing about how disease affects the more practical, relational or existential aspects of life as experienced by the patients themselves, nor does it consider the psychosocial resources that patients need to cope with in their daily life. Research has shown that physicians tend to underestimate their patients’ health status [20] and to consider that side effects must be medical and life-threatening in order to be regarded as harmful. However, patients may blame ‘the pill’ for symptoms or challenges that are not necessarily related to their medication. For example, one patient reported being less happy as a side effect of imatinib. Whether this lack of happiness is a side effect, a consequence of the stressful situation of living with a chronic cancer or some other issue remains uncertain. As pointed out by Poort et al., fatigue is disabling and not only associated with current TKI use, but also with psychological distress and physical functioning [12]. Nevertheless, we hypothesise that challenging and debilitating side effects, as experienced by patients with metastatic GIST, may not be noticed and given appropriate attention. During follow up, it seems important that the oncologist develops a comprehensive picture of what it means for the patients to live with metastatic GIST. The subjective and psychosocial consequences of the treatment should be captured, and communication on how to deal with this should be an essential part. However, it is not necessarily the oncologist who should be responsible for the supportive care. Cancer nurses, other health care professionals and peers might play important roles. Improved communication might help the patients to better cope with the side effects that negatively impact their everyday life. Further research on how daily practical and psychosocial life is affected by the subjective side effects of cancer treatment, as well as how best help to patients to overcome those daily challenges, is hence warranted. Also, actively exploring dose reduction of imatinib tailored to the patients’ side effect and disease control, as well as interventions like cognitive behavioural therapies, might be justified.

The majority of our participants described ‘a new normal’ involving new preconditions whereby they experienced challenges, such as tiredness and fatigue, as well as a reduction in work capacity and social life, when compared to their ‘old normal’. From a psychosocial perspective, serious disease can be understood as a biographical disruption and a serious incident in a person’s life. Their previous life story is disrupted, as is their identity [14, 21]. Our participants described how illness had broken into their daily life and altered their life experiences. This experience of ‘otherness’ can lead to a sense of standing out negatively from the perceived norm, as well as to a reduced social life [21]. Most of our participants were very keen to lead a life as normal as possible. Attempting to continue with the activities and social relationships that correspond to their pre-disease life is a feature of the biographical disruption seen in chronically ill people. However, their sense of normality had to be changed and adapted to the new constraints in their life. This appeared particularly evident when the participants expressed the wish to return to the roles they had before the disease struck [21]. Existing in a world of disease can be a challenging situation. Striving for normality in the form of a new normal can, for chronic GIST patients, represent a means of coping with physical and existential challenges and distancing oneself from the disease. It is hence vitally important that sufficient attention is being paid to this issue during the follow-up of such patients.

The fact that several of our participants emphasised that they considered themselves to be healthy, despite having metastatic cancer and experiencing side effects of the associated treatment, could be related to questions such as ‘what is health and when is a person healthy?’ An individual’s ability to cope and to achieve vital goals is more important than whether that individual is defined as healthy or ill. In fact, the articular-holistic health concept stresses that health must be linked to action and function [22]. This view contradicts the prevailing notion of health as the absence of disease. In addition to being able to achieve vital goals and maintain everyday activity, health involves one’s ability to accomplish education and to work, to lead an active and social life, to have the opportunity to form close relationships and to establish a family, that is, living a normal life. In line with the findings of this study, this may be of particular relevance to those living with metastatic GISTs and, therefore, fighting everyday challenges.

Health is a way of being in the world. It is concerned with being in a meaningful relationship with others [23]. It has been emphasised that one cannot divide health into only somatic and psychological issues, since health is part of a being’s completeness (the whole of being), where we find our own well-being in the world in which we live. The prevailing paradigm in modern medicine has a unilateral focus on science and technology that may counteract the possibility to see the whole person, including that person’s health challenges and opportunities [23]. For chronic GIST patients, this means that cancer treatment is a prerequisite for extended survival, although it may not be sufficient.

This study did have certain limitations. As with most qualitative studies, the small sample size limits its generalisability. Nevertheless, the obtained narratives are rich and full of nuanced examples. In qualitative research, one does not seek representative data, but rather aims to illuminate the phenomena that participants experience from their own perspectives.

## Conclusions

Advances in cancer research that result in new treatments will, over the next few decades, probably result in more patients with different cancer diagnoses living in a chronic phase, which means that they cannot be cured, although they will remain in stable remission for several years. This should prompt more attention and research concerning how daily practical and psychosocial life is affected in patients with metastatic cancer who are in long-term remission. In this regard, metastatic GIST could serve as a model disease due to the high response rate and relatively long expected survival in these patients. Our findings indicate that, for those patients who are living in limbo between having metastatic cancer and receiving effective treatment, a holistic view of health on the part of healthcare providers is crucial. One vital goal should be to improve communication between healthcare professionals and cancer patients in order to secure a comprehensive follow-up with guidance on how to cope with, and adapt to, their new normal.

## Additional file

**Additional file 1.** Interview schedule.

## Abbreviations

GIST: gastrointestinal stromal tumour
NRH OUH: Norwegian Radium Hospital, Oslo University Hospital
TKI: tyrosine kinase inhibitors
QoL: quality of life
