# Supplementary material for: Perspectives on treatment side effects in patients with metastatic gastrointestinal stromal tumour: a qualitative study
Source: Clin Sarcoma Res. 2019 Apr 30;9:6. doi: 10.1186/s13569-019-0116-3 (PMC6492319; doi:10.1186/s13569-019-0116-3)
Supplement: Supplementary file 1 — Additional file 1. Interview schedule. [file 13569_2019_116_MOESM1_ESM.docx]

**Interview schedule**

Patients with chronic GIST

(translated from Norwegian)

Could you tell me your entire cancer story?

- From the time you got sick
- When you got the diagnosis
- About treatment experiences
- Until your current situation

How do you experience living with chronic cancer?

What are the physical and practical consequences of the disease and treatment?

What is the impact of the disease and treatment in your everyday life?

How has living with chronic cancer affected you as a person?

Could you tell me about your relationship regarding “the pill”?

Do you consider yourself to be healthy or ill? Why…..?

Something more you want to share?
